# Postoperative Pneumocephalus on Computed Tomography Might Predict Post-Corpus Callosotomy Chemical Meningitis

**DOI:** 10.3390/brainsci11050638

**Published:** 2021-05-15

**Authors:** Ayataka Fujimoto, Keisuke Hatano, Toshiki Nozaki, Keishiro Sato, Hideo Enoki, Tohru Okanishi

**Affiliations:** Comprehensive Epilepsy Center, Seirei Hamamatsu General Hospital, 2-12-12 Sumiyoshi, Nakaku, Hamamatsu, Shizuoka 430-8558, Japan; hatakenosuke@gmail.com (K.H.); tonod03sm069@gmail.com (T.N.); k-sato@sis.seirei.or.jp (K.S.); enokih.neuropediatr@gmail.com (H.E.); t.okanishi@tottori-u.ac.jp (T.O.)

**Keywords:** corpus callosotomy, chemical meningitis, pneumocephalus, high fever, risk factor

## Abstract

Background: A corpus callosotomy (CC) is a procedure in which the corpus callosum, the largest collection of commissural fibers in the brain, is disconnected to treat epileptic seizures. The occurrence of chemical meningitis has been reported in association with this procedure. We hypothesized that intraventricular pneumocephalus after CC surgery represents a risk factor for postoperative chemical meningitis. The purpose of this study was to analyze the potential risk factors for postoperative chemical meningitis in patients with medically intractable epilepsy who underwent a CC. Methods: Among the patients who underwent an anterior/total CC for medically intractable epilepsy between January 2009 and March 2021, participants were comprised of those who underwent a computed tomography scan on postoperative day 0. We statistically compared the groups with (c-Group) or without chemical meningitis (nc-Group) to determine the risk factors. Results: Of the 80 patients who underwent a CC, 65 patients (25 females and 40 males) met the inclusion criteria. Their age at the time of their CC procedure was 0–57 years. The c-Group (17%) was comprised of seven females and four males (age at the time of their CC procedure, 1–43 years), and the nc-Group (83%) was comprised of 18 females and 36 males (age at the time of their CC procedure, 0–57 years). Mann–Whitney U-tests (*p* = 0.002) and univariate logistic regression analysis (*p* = 0.001) showed a significant difference in pneumocephalus between the groups. Conclusion: Postoperative pneumocephalus identified on a computed tomography scan is a risk factor for post-CC chemical meningitis.

## 1. Introduction

The corpus callosotomy (CC) procedure was introduced in 1940 by van Wagenen and Herren for patients with epilepsy secondary to glioma [1,2]. This disconnective surgical technique is applied for the treatment of intractable epileptic seizures [3,4,5,6]. The corpus collosum is the largest collection of commissural fibers in the brain [7]. To perform CC surgery, we have the options of open cranial surgery [8], laser thermal [9], radiosurgical [10], and endoscopic approaches [11,12] to disconnect the corpus callosum. A callosal transection is performed between the two leaves of the septum pellucidum at the midline cleft and the ependyma of the lateral ventricles as the inferior edge of the disconnection [13]. Iatrogenic meningitis has been reported in association with this procedure [12,14,15,16]. This meningitis is probably induced by intraventricular inflammation if the ependymal membrane is breached during the disconnection of the corpus callosum. A previous report showed that the frequency of chemical meningitis after CC surgery was >33% [17]. Prophylactic corticosteroids can thus be used to prevent chemical meningitis following CC surgery [5]. However, not all facilities use steroids perioperatively, including our facility [18]. Due to advances in modern medicine, such as microscopic surgery and artificial cerebrospinal fluid (CSF), we do not always experience chemical meningitis at a rate as high as >33%. Since the procedure for a CC differs from hemispherical to lobe disconnection surgery, which requires a large opening in the lateral ventricle [19,20], rupture of the ependymal membrane during CC surgery is sometimes not obvious and might be missed. We sometimes found that if we opened the ependymal membrane, postoperative pneumocephalus was identified in computed tomography (CT) images. We therefore thought that postoperative chemical meningitis might be predictable based on a finding of postoperative pneumocephalus in CT images. In this study, we hypothesized that intraventricular pneumocephalus after CC surgery represented a risk factor for postoperative chemical meningitis. The purpose of this study was to analyze the frequency of postoperative chemical meningitis and potential risk factors for patients with medically intractable epilepsy who underwent CC surgery.

## 2. Methods

### 2.1. Study Design and Ethics Approval

The ethics committee at Seirei Hamamatsu General Hospital, Japan, approved the protocol for this retrospective study (approval no. 3590), which was performed in accordance with the principles of the Declaration of Helsinki. Subjects in this study were identified from a review of the electronic medical records for patients who had undergone epilepsy surgery between February 2019 and March 2021 in the Comprehensive Epilepsy Center at Seirei Hamamatsu General Hospital.

### 2.2. Clinical Information

We performed an anterior or a total CC for patients with medically intractable epilepsy between January 2009 and March 2021 in our hospital. Among these patients, those who underwent a CT scan on postoperative day 0 were enrolled and patients who fulfilled the following criteria were excluded: (1) postoperative complications such as intracerebral hematoma, subdural or epidural hematoma, pneumonia, urinary tract infection, etc.; (2) tube feeding required due to a postoperative disturbance of consciousness [8]; or (3) antibiotics required for bacterial meningitis. The reason we excluded patients who required tube feeding was that micro-aspiration might have caused bronchitis or pneumonia [21].

### 2.3. Corpus Callosotomy

Under general anesthesia, the patient was positioned supine on the operating table with the head fixed in a Mayfield head clamp. The head position of the patient was registered to a neuronavigation system (iPlan Cranial software; Brainlab AG, Feldkirchen, Germany). We made a small bicoronal skin incision around the bregma. A craniotomy was performed using four burr holes, placed 3 cm anterior, 2 cm posterior, 3 cm to the right, and 3 cm to the left of the bregma, then a wide U-shaped dural flap was made on either side to avoid a cortical vein injury. Under microscopy, retractor blades were placed over the cortex and falx, exposing the inferior falx and cingulate gyri. The interhemispheric fissure was divided to reach the corpus callosum. The callosal division was initiated with two microsuction tubes at the level of the body of the corpus callosum. After an anterior/total CC, the trajectory of the interhemispheric fissure was rinsed with 500 mL of artificial CSF followed by a dura mater closure, bone flap repositioning, and skin closure.

### 2.4. Diagnosis of Chemical Meningitis

We regarded the following as criteria for chemical meningitis: (1) an acute-onset high fever (≥39 °C) starting on the postoperative day (POD)0–1; (2) lasting ≤7 days; (3) no apparent wound infection; (4) pneumonia or urinary tract infection ruled out using a chest X-ray, urine examinations, and urinary culture; and (5) no need for additional antibiotics [22,23]. All patients were regularly administered cefazolin, with a dose of 20 mg/kg for those patients with a bodyweight of <50 kg and 1 g/kg for those with a body weight of ≥50 kg, diluted with 50 mL of saline within 30 min of making the initial skin incision. We also administered the same dose of cefazolin 6 h after the first administration [24]. We did not always perform a lumbar tap for the CSF examinations because postoperative results for CSF with a high white blood cell count might not provide consistent information to diagnose chemical meningitis. We did not perform a CSF culture for the patients who met the criteria for chemical meningitis. Patients who exhibited a gradual onset fever (≥3 days after CC surgery) were regarded as having possible bacterial meningitis and additional antibiotics were used; such patients were excluded from this study. Regarding a high-grade fever, because patients received anti-inflammatory medications such as aspirin or corticosteroid, we counted the consecutive days that patients had a temperature ≥39 °C. As some patients showed nuchal rigidity after open cranial surgery and some patients did not, we did not review postoperative nuchal rigidity in this study.

### 2.5. Postoperative Pneumocephalus 

CT scans were performed postoperatively for all patients who underwent CC surgery to detect the air density (<−1000 Hounsfield units [HU]) in the lateral ventricles. As we routinely perform CT scans for image guidance just before [25] and after CC surgery in the operation theater, we regarded the new appearance of a low-density area in a lateral ventricle as pneumocephalus.

### 2.6. Outcome Measurement

We divided the patients into a group that exhibited chemical meningitis (c-Group) and a group that did not exhibit chemical meningitis (nc-Group). We compared these two groups statistically. To identify the risk factors for chemical meningitis, we used univariate logistic regression analyses to analyze the correlations between chemical meningitis and potential predictors.

The operation time, blood loss, and anterior/total CC were also reviewed. We reviewed the number of consecutive days that patients had a temperature ≥ 39 °C in the c-Group.

### 2.7. Statistical Analysis

Comparisons between the groups were made using Mann–Whitney U-tests and univariate logistic regression tests. As the number of events of chemical meningitis was too small to allow the application of multivariate analysis, we only performed univariate logistic regression analysis [26,27,28]. Values of *p* < 0.05 were considered to indicate significant differences in all analyses. All statistical analyses were performed using Sigma Plot version 14.0 software (Systat Software, San Jose, CA, USA).

## 3. Results

### 3.1. Clinical Information

Of the 80 patients who underwent CC surgery, the 65 that met the study’s criteria comprised of 25 females and 40 males with an age at the time of their CC surgery ranging from 0 to 57 years (mean, 15.7 years; standard deviation [SD], 14.8 years; median, 9 years). Of these, the c-Group was comprised of 11 patients (17%; seven females and four males) with an age at the time of their CC surgery ranging from 1 to 43 years (mean, 11.2 years; SD, 12.3 years; median, 8 years), and the nc-Group was comprised of 54 patients (83%; 18 females and 36 males) ranging in age from 0 to 57 years (mean, 16.6 years; SD, 15.2 years; median, 12 years) (Table 1).

### 3.2. Outcome Measurements

The univariate logistic regression analysis showed a significant difference in the frequency of pneumocephalus between the groups (*p* = 0.001). Other factors, including the operation time and blood loss, are shown in Table 2. The number of consecutive days on which patients had a fever ≥39 °C in the c-Group ranged from 1 to 5 days (mean, 1.64 days; SD, 1.28 days; median, 1 day).

### 3.3. Representative Case of Chemical Meningitis

**Figure 1:**
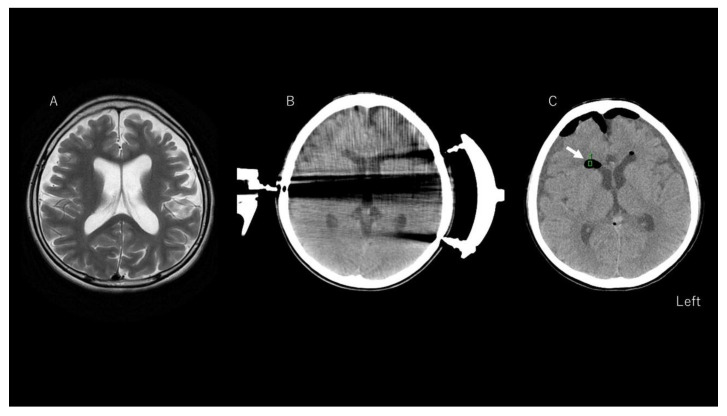
Results of neuroimaging in a representative case (Patient 56). T2-weighted imaging shows no structural lesions except for an atrophic appearance of the brain compared to norms for the patient’s age (**A**). A preoperative computed tomography (CT) image of the brain shows no intraventricular air density (**B**), even though halation artifacts from the Mayfield head holder are seen (this CT image was taken preoperatively for image guidance). A postoperative CT image (**C**) shows low density in the lateral ventricle (see arrow) representing pneumocephalus. The green square is the region of interest to calculate CT density (<−1000 HU).

Patient 56, a 12-year-old boy with Lennox–Gastaut syndrome, had suffered from epileptic spasms since he was one year and three months old, and currently had multiple types of seizures despite ongoing treatment with phenobarbital, valproic acid, zonisamide, lamotrigine, levetiracetam, topiramate, gabapentin, clonazepam, clobazam, and rufinamide for more than 10 years. As he experienced head and facial trauma from daily tonic seizures, and also experienced status epilepticus every 2–3 months, he was referred to our hospital for a total CC. Magnetic resonance imaging of the brain showed an atrophic brain for his age. A postoperative CT scan of the total CC showed pneumocephalus (Figure 1). Postoperatively, his body temperature reached 40 °C, and his temperature was ≥39 °C for three consecutive days. He was given aspirin, but not corticosteroid. The patient was discharged on postoperative day 15 without any neurological complications. After the CC surgery, the frequency and intensity of his tonic seizures improved to monthly, with no further episodes of status epilepticus.

## 4. Discussion

Pneumocephalus was the only risk factor supported by all statistical analyses among the evaluated factors. The mechanisms of chemical meningitis have not been adequately elucidated. However, pneumocephalus was found to be associated with chemical meningitis in this study. As the process by which pneumocephalus results from the rupture of the ependymal membrane during surgical procedures is natural, the cause of chemical meningitis is probably related to the entry of air or artificial CSF into the ventricle. Even though the exact mechanisms by which the air or artificial CSF entering the ventricle creates the pathophysiology of chemical meningitis remain unclear, since chemical meningitis can also result from conditions such as a ruptured dermoid cyst [29], a craniopharyngioma [30,31], and an intraventricular hemorrhage [32], inflammation of the central nervous system appears related to chemical meningitis [33] because neutrophilic pleocytosis has been experimentally shown [34].

Mann–Whitney U-tests showed significant differences in sex and blood loss between the c-Group and the nc-Group. If these findings were correct, a smaller blood loss would be predicted to be associated with a greater probability of chemical meningitis. These outcomes were therefore considered to be statistically paradoxical results. The significance of sex might be due to the greater number of males enrolled in this study.

Even though the presence of postoperative pneumocephalus in CT images after CC surgery may predict the occurrence of chemical meningitis with a 70% probability, whether we should use steroid prophylactically could not be determined from this study. 

At the very least, pneumocephalus as a potential risk factor for chemical meningitis after CC surgery should be considered as a warning sign to prepare for the possibility of postoperative chemical meningitis, even though postoperative meningitis still constitutes a diagnostic and therapeutic challenge in patients after open cranial surgery.

Further research is needed to compare groups with or without prophylactic steroid use after CC surgery.

## 5. Conclusions

Postoperative pneumocephalus in CT imaging is a risk factor of post-CC chemical meningitis. In this study, since we did not evaluate the prophylactic use of steroids, comparisons between groups with or without the prophylactical use of steroids are warranted as future research.

## Figures and Tables

**Table 1:** Clinical information.

	c-Group (*n* = 11; 17%)	nc-Group (*n* = 54; 83%)	*p*-Value
Age, y	mean 11.2, SD 12.3, median 8	mean 16.6, SD 15.2, median 12	0.244
Sex	seven females, four males	18 females, 36 males	0.016 *
Body mass index, kg/m^2^	mean 16.3, SD 3.23, median 15.5	mean 19.1, SD 5.11, median 17.6	0.07
Operation time, mins	mean 177.9, SD 75, median 153	mean 208.1, SD 77.6, median 173.5	0.159
Blood loss, mL	mean 31.2, SD 40.6, median 20.0	mean 66.2, SD 75.0, median 30.0	0.041 *
Pneumocephalus	8 (73%)	13 (24%)	0.002 *
Surgery (total CC)	3 (27%)	25 (44%)	0.556

CC, corpus callosotomy; SD, standard deviation. * *p* < 0.05, Mann–Whitney U-tests were applied.

**Table 2:** Correlations between chemical meningitis and predictors.

	Coefficient	Standard Error	*p*-Value
Age	−0.003	0.003	0.270
Sex	0.180	0.090	0.061
Operation time	−0.001	0.001	0.242
Blood loss	−0.001	0.001	0.139
Pneumocephalus	0.313	0.093	0.001 *
Total vs. Anterior CC	0.109	0.094	0.252

CC, corpus callosotomy. * *p* < 0.05.

## Data Availability

The data are not publicly available due to patients’ privacy.
